# Management of liver trauma in urban university hospitals in India: an observational multicentre cohort study

**DOI:** 10.1186/s13017-020-00338-9

**Published:** 2020-10-15

**Authors:** Yash Sinha, Monty U. Khajanchi, Ramlal P. Prajapati, Satish Dharap, Kapil Dev Soni, Vineet Kumar, Santosh Mahindrakar, Nobhojit Roy

**Affiliations:** 1 Gastrointestinal Surgery, Sanjay Gandhi Post Graduate Institute, Rai Bareily Road, Lucknow, Uttar Pradesh 226014 India; 2 Seth GSMC and KEM Hospital, Dr E Borges Road, Mumbai, 400012 India; 3 Topiwala National Medical College & B.Y.L. Nair Charitable Hospital, Mumbai, 400008 India; 4 JPN Apex Trauma Center, All India Institute of Medical Sciences, Raj Nagar, Safdarjung Enclave, New Delhi, 110029 India; 5 LTMMC & LTMG Hospital, Sion, Mumbai, India; 6 Innovative Alliance for Public Health, New Delhi, H. No 87F, H Block, Phase 6, Taj Avenue, Ayanagar, New Delhi, 110047 India; 7 Department of Global Public Health, Karolinska Institutet, Tomtebodavägen 18 A, Widerströmska Huset, SE-171 77 Stockholm, Sweden; 8 WHO Collaborating Centre for Research on Surgical Care Delivery in LMICs, Department of Surgery, BARC Hospital (Govt. of India), Mumbai, 400094 India

**Keywords:** Injury, Liver injury, Non-operative management, Epidemiology of liver injury, Management

## Abstract

**Background:**

Low- and middle-income countries (LMICs) contribute to 90% of injuries occurring in the world. The liver is one of the commonest organs injured in abdominal trauma. This study aims to highlight the demographic and management profile of liver injury patients, presenting to four urban Indian university hospitals in India.

**Methods:**

This is a retrospective registry-based study. Data of patients with liver injury either isolated or concomitant with other injuries was used using the ICD-10 code S36.1 for liver injury. The severity of injury was graded based on the World Society of Emergency Surgery (WSES) grading for liver injuries.

**Results:**

A total of 368 liver injury patients were analysed. Eighty-nine percent were males, with road traffic injuries being the commonest mechanism. As per WSES liver injury grade, there were 127 (34.5%) grade I, 96 (26.1%) grade II, 70 (19.0%) grade III and 66 (17.9%) grade IV injuries. The overall mortality was 16.6%. Two hundred sixty-two patients (71.2%) were managed non-operatively (NOM), and 106 (38.8%) were operated. 90.1% of those managed non-operatively survived.

**Conclusion:**

In this multicentre cohort of liver injury patients from urban university hospitals in India, the commonest profile of patient was a young male, with a blunt injury to the abdomen due to a road traffic accident. Success rate of non-operative management of liver injury is comparable to other countries.

## Introduction

Injuries account for 4.8 million lives globally, and deaths due to road traffic injuries alone are among the top 10 causes of mortality [1, 2]. Seven to ten percent of all injuries that occur involve the abdominal region, making it the third most common region injured following traumatic brain injury (TBI) and extremity injury [3, 4]. Liver and spleen injuries are the commonest damages in blunt abdominal trauma [5].

Promising outcomes of non-operative management (NOM), in paediatric splenic injuries, have shifted the definitive treatment of these injuries from operative management (OM) to NOM [6, 7]. Higher grade injuries to the liver can be conserved if the patient is hemodynamically stable [8, 9]. NOM is based on the understanding that an injury which appears severe may not necessarily exsanguinate and haemostasis does occur naturally, at least in some cases. NOM is now possible because of multidetector computerised tomography (CT) scan, intervention radiology and intensive care monitoring along with a paradigm shift in the concept of haemostasis [10]. This has decreased the mortality and morbidity in patients with high-grade liver trauma. OM of liver injury is only considered for those who are hemodynamically unstable or if NOM fails [8].

LMICs like India contribute to 90% of all the global injury burden, which is a critical public health issue [11]. Most published literature from India is anecdotal or single-centre studies with small database [12–16]. A multicentre hospital-based registry can help in better understanding the outcomes in the management of organ-specific injuries. In 2013, a four-university hospital registry study, called Towards Improved Trauma Care Outcomes in India (TITCO), was initiated to observe the demography, injury aetiology, management and outcomes of injured patients in urban India [17]. The aim of this study is to conduct a subgroup analysis of patients with liver injuries, managed in one such a large multicentre hospital-based registry in urban India.

## Methods

### Study design

This is a retrospective registry-based study with data extracted from a prospective cohort study called Towards Improved Trauma Care Outcomes in India (TITCO). The TITCO study is a multicentre research consortium of university hospitals formed to develop a trauma registry in India.

### Setting

The study was conducted in four public university hospitals in India between October 2013 and December 2015. The hospitals included in the study are from three metropolitan cities, namely Mumbai, Delhi and Kolkata. The hospitals were King Edward Memorial Hospital (KEMH) and Lokmanya Tilak Municipal General Hospital (LTMGH) in Mumbai, Jai Prakash Narayan Apex Trauma Centre (JPNATC) in New Delhi and the Institute of Post-Graduate Medical Education and Research and Seth Sukhlal Karnani Memorial Hospital (SSKM) in Kolkata.

The urban referral trauma centres are situated in Kolkata, Mumbai (2 centres) and Delhi, cities with populations of more than 10 million. Except for the JPNATC, which is a standalone trauma centre, the others are trauma units providing trauma care as a part of a general hospital. The user fees are nominal and classified as free to public. The hospitals mainly serve the lower socioeconomic strata of the population in their respective area. Each of these hospitals receives 40 to 100 major trauma patients per week. They have round the clock emergency services, imaging, operating theatres and sub-speciality available.

### Source and method of participant selection

All admitted patients that presented with history of trauma on arrival to any of the study hospitals were included in the TITCO registry. Data of patients with liver trauma either isolated or concomitant with other injuries was extracted using the ICD-10 code S36.1 for liver injury.

### Data collection

Project officers included those with a master in science, who were then trained in the methods of data selection for the study in a workshop format, for a period of 1 week. These trained project officers at each hospital worked 8-h shifts with a rotating schedule between day, evening and night shifts through all days of the week. Data from patients admitted outside of the shift hours was collected retrospectively from the hospital medical records. The patients were followed up until discharge, death or to a maximum of 30 days. If discharged before 30 days, the patients were considered to be alive at 30 days. There was no follow-up after patient discharge or after the 30 days.

### Study variables

The primary outcome was 30-day in-hospital mortality following liver injury. Patients who died during their hospital stay up to 30 days were recorded. Those discharged before 30 days were considered to be alive at 30 days. The data set was analysed for patients’ demographic profile, mechanism of injury, severity, management and outcome.

The data also included serially recorded parameters like pulse, systolic blood pressure (SBP), Glasgow Coma Score (GCS) and interventions done, if any. Those patients with a systolic blood pressure of ≤ 90 mmHg were considered as hemodynamically unstable having hypotension.

The severity of injury has been graded based on the World Society of Emergency Surgery (WSES) guidelines. WSES grading of liver injuries has been graded based on the American Association of Surgery for Trauma (AAST) scale (anatomical classification of liver injuries) and the hemodynamic stability (physiological parameter) for grading liver injuries from I-IV [8]. The classification has been added as an additional file (see Additional file 1). Management of liver injury in these four centres was not as per the WSES guidelines for liver trauma. WSES liver injury grades were first published in 2015, by which time the participating centres finished data collection.

Patients’ management was divided and labelled as operative management (OM) in those who underwent laparotomy and NOM in those who were conservatively managed without a laparotomy. Those patients who survived NOM were labelled as successfully managed. The patients who died after NOM were labelled as NOM failure. The overall management of these patients along with the treatment for other associated injuries was recorded.

### Quantitative variables

All continuous variables were represented as mean with their standard deviation and categorical variables as counts and proportions. ISS was represented as median with inter-quartile range.

## Results

### Demographics and liver trauma profile (Table 1)

Out of the 16,047 trauma patients in the TITCO registry, 1134 (7.1%) patients suffered abdominal trauma, of which 368 (32.5%) had liver trauma. Age range varied between 2 and 80 years with the mean age of 26 years with 328 (89%) being males. The main mechanism of injury was road traffic injury (RTI) accounting for 57% of the patients. Among the RTI, the largest group were motorcyclist injuries (30.48%). More than half of the patients were transferred patients from other referral centres (58.2%). 91.5% of the cohort with liver injuries had blunt injuries. Eighty-eight (24.5%) patients presented on arrival with SBP of ≤ 90 mmHg.

**Table 1:** Demographics and clinical profile of patients with liver injury

Variables	Value (*n* = 368)	Missing values (*n*)
Age	26 (12.7)	0
Male, *n* (%)	328 (89%)	0
Mechanism of injury		2
1. Road traffic accident	210 (57.07%)	
2. Railways	24 (6.52%)	
3. Assault	39 (10.6%)	
4. Falls	79 (21.47%)	
5. Other	14 (3.8%)	
Blunt injury	337 (91.6%)	0
Heart rate (beats per minute)	99 (19.3)	8
Systolic BP (mmHg)	108 (23.4)	9
Haemoglobin, g/dl (mean ± SD)	11 (2.1)	21
ISS score, median (IQR)	17 (10-22)	
GCS score	13.4 (3.3)	11
Length of stay, in days, median (IQR)	8.5 (4.8-15.0)	2
Units of blood received in those operated (mean)	1.8 units (2.0)	–
WSES liver injury grade		9
I	127 (34.5%)	
II	96 (26.1%)	
III	70 (19.0%)	
IV	66 (17.9%)	
NA (as SBP missing)	9 (2.4%)	

Continuous variables are represented by mean with their standard deviation in parentheses except for ISS and length of stay where they are shown as median and IQR. Categorical variables are represented as counts and proportions in parenthesis

**Fig. 1:**
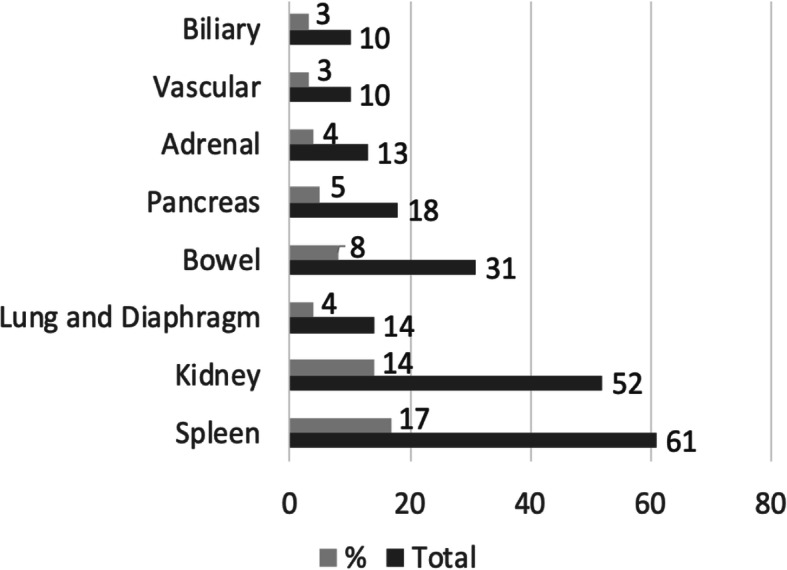
Number and proportions of other abdomino thoracic injuries along with liver injuries

Most of liver trauma patients belonged to WSES grades I-III (75%). Nine patients could not be classified as WSES grade as their systolic blood pressure was missing. The most common intra-abdominal injuries associated with liver trauma were spleen (17%) and kidney (14%) (Fig. 1). Eighty-five patients had an associated TBI of which 38 (44.7%) had moderate to severe TBI based on GCS.

### Management and outcome in liver injury (Fig. 2)

#### Diagnostic modalities

Focused Assessment with Sonography in Trauma (FAST) was done in 345 patients (93.8%), and a CT scan was done in 310 (84.24%) patients included in the study.

**Fig. 2:**
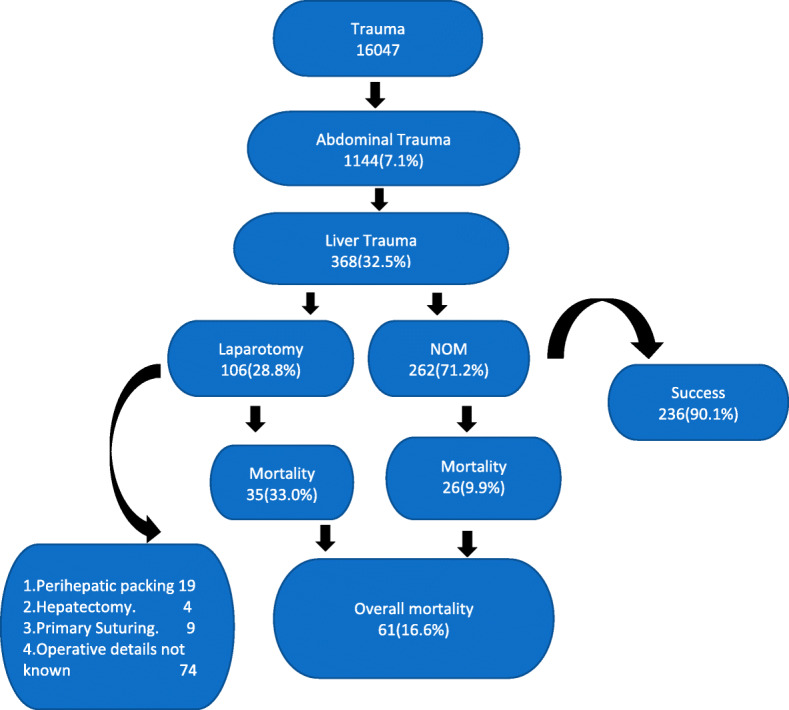
Management and outcomes in liver injury patients

#### Overall mortality

Overall, 30-day in-hospital mortality rate in this cohort of liver injury with/without other injuries was 16.6% (61 out of 368).

#### Non-operative management

Out of 368 patients with liver trauma and other associated injuries, 262 (71.2%) patients had NOM. The NOM as per various WSES grade of liver injury is shown in Table 2. Among these, 236 patients (90.1%) were successfully managed (survived) (Fig. 2). As per the WSES grades of injury, the NOM success rates were grade I—90.2%, grade II—90.6%, grade III—93.1% and grade IV—81.6%.

**Table 2:** Operative and non-operative management of liver injuries as per WSES grade of liver injury

WSES grade of liver injury	NOM (%)	OM for liver ± other intra-abdominal organ (a)	OM for intra-abdominal organ other than liver (b)	OM for unspecified reason (c)	Total no. of operated (a + b + c) (%)
I	102 (39.0)	7	13	5	25 (23.6)
II	64 (24.4)	11	16	5	32 (30.2)
III	58 (22.1)	6	4	2	12 (11.3)
IV	33 (12.6)	18	11	4	33 (31.1)
NA	5 (1.9)	1	1	2	4 (3.8)
Total	262	43	45	18	106

*WSES* World Society Emergency Surgery, *NOM* non-operative management, *OM* operative management

Death occurred in 26 patients (9.9%). Four of them died within 24 h of arrival, 11 died between 24 h and 7 days after arrival and 11 died after 7 days from arrival (time data of one patient was missing). Of those who died, 7 patients had severe TBI (< 8 GCS and intracranial injuries), 5 patients had mild TBI (> 12 GCS), 2 patients had hypotension and TBI, 5 patients had hypotension without TBI and seven had no hypotension on arrival and no TBI.

#### Operative management

One hundred six patients underwent emergency laparotomy which included various procedures such as packing both perihepatic and intraparenchymal haemostatic packs, direct suture ligation of lacerations, anatomic or nonanatomic segmental hepatectomy for liver injury, splenectomy, nephrectomy and bowel suturing for associated injuries. Of the 106 laparotomies, 43 (40.5%) were for liver and/or other intra-abdominal organs, 45 (42.5%) were for other intra-abdominal organs only (non-liver reasons) and 18(17.0%) were for cause unspecified (Table 2). Twenty-two (20.8%) patients with penetrating injury underwent OM. Of these 22 penetrating injuries, 3 patients died. Among the operated 106 patients, 13 patients (12%) were taken to the operating room within 1 h of admission while the rest underwent surgery within 24 h of admission. Forty-six (43.4%) patients did not get a CT scan done before surgery.

As per the WSES grades of injury, of those who underwent OM, 25 (23.6%) had grade I, 32 (30.9%) were grade II, 12 (11.3%) were grade III and 33 (37.7%) were grade IV liver injuries. Of the 69 grade I-III liver injury patients, 24 were operated for liver and/or other associated intra-abdominal organ injury, of which 8 had penetrating injury. The rest 45 were operated for other intra-abdominal organ injury (non-liver) or for unspecified reasons (Table 2).

The operative management cohort differed from the non-operative cohort significantly in their mean SBP, 99 (26.2%) vs. 111 (21.0%); proportion of penetrating injury, 21.7% vs. 4.4%; heart rate, 103 (2.4) vs. 97 (18.5); and ISS, 14 (9-22) vs. 17 (12-22). Univariate analysis showed no difference between these two cohorts in their age and GCS (Table 3). The injury severity score (ISS) in the NOM group was higher, compared to those who underwent laparotomy. One third of the patients who underwent laparotomy died (35 out of 106). The causes of death in these patients cannot purely be assigned to liver trauma as they had multiple injuries. Fourteen of them died within 24 h of arrival, 16 died between 24 h and 7 days after arrival and 8 died after 7 days (time data of one patient was missing).

**Table 3:** Comparison of physiological variables of patients who underwent laparotomy vs. those who underwent non-operative management

Variables	OM (*n* = 106)	NOM (*n* = 263)	*p* value
Age	28 (12.8)	25 (12.6)	*p* = 0.07 *t* test
Penetrating injury (%)	22 (20.8)	9 (3.5)	*p* < 0.05 *t* test
SBP (mmHg), mean (SD)	99 (26.6)	111 (21.0)	*p* < 0.05 *t* test
Heart rate (beats per minute), mean (SD)	103 (20.4)	97 (18.5)	*p* < 0.05 *t* test
GCS, mean (SD)	13 (3.9)	14 (3.1)	*p* = 0.14 *t* test
ISS, median (IQR)	14 (9-22)	17 (12-22)	*p* < 0.05 (Wilcoxon’s rank-sum test)
Mortality, *n* (%)	35 (33.0%)	26 (9.9%)	*p* < 0.05 chi-square

*SBP* systolic blood pressure, *GCS* Glasgow Coma Scale, *OM* operative management (laparotomy), *NOM* non-operative management, *ISS* injury severity score

## Discussion

To our knowledge, this is the first analysis of an Indian multicentre cohort of liver injury patients and has one of the largest cohorts analysed in India and probably across LMICs.

In our study, a third of all the abdominal trauma patients had liver injury. More than half were RTI, and the majority were blunt type of injury to the abdomen. In our study, the proportion of liver injuries within the abdominal region was 33% and is similar to other studies from India which reported 23-35% of all the abdominal injuries [18–20]. However, this is lower than the proportion of 42-52% reported from studies from Africa and Italy [4, 21]. In India, blunt abdominal trauma due to RTI is the commonest mechanism of injury except in the state of Jammu and Kashmir (a conflict zone) which has a higher proportion of penetrating abdominal trauma [19]. In countries where assault is common, penetrating injuries are the most common cause of abdominal injury thence liver injuries [22–24].

The mean age was 26 years with a predominance of males (89%). This could be as liver injury occurs most commonly in young adults who extensively travel for work and engage in sporting activities compared to women [25]. Our cohort also reflects this, with RTIs being more common in males, compared to females who predominantly have falls. Consequently, liver injuries are common in males.

In our cohort of liver injury patients, 90-93% of the WSES grade I-III liver injuries were successfully managed using non-operative management (NOM) strategy. In WSES grade IV liver injuries, this number of NOM success was reduced to 84%. Progress in the management of liver trauma towards the end of the twentieth century has reduced the mortality [7]. Serial imaging, advancements in critical care and adjunctive therapies like angiography, percutaneous drainage and endoscopy/endoscopic retrograde cholangiopancreatography management of hepatic injuries have resulted in improved outcomes [9]. Literature suggests most liver injuries of grades I-III are treated by NOM with 82-100% success [9, 26, 27]. However, studies comparing OM vs. NOM in high-grade liver injury are still evolving [28]. Our comparisons of the two cohorts showed poor outcomes in those undergoing OM. On admission, the OM cohort had poor physiological variables compared to NOM, suggesting this cohort to have more serious injuries. Median ISS of OM cohort (ISS = 14) was significantly less compared to that of NOM cohort (ISS = 17). ISS is a poor predictor of severity in LMICs. This has been repeatedly demonstrated in predictor studies on mortality in trauma [29, 30].

In our study, approximately 50% of WSES grade IV liver trauma were managed non-operatively. This is unlike the guidelines and other literature where such kind of injuries would have been operated [9, 31]. In our study, we have classified them as WSES grade IV based on the on-arrival systolic blood pressure < 90 mmHg and any grade of injury. Systolic blood pressure is a dynamic process and changes as the patient is being resuscitated. In our opinion, the reasons for non-operative management of these grade IV liver injuries may have been due to (i) improvement of systolic blood pressure after resuscitation (responders or borderline unstable patients), (ii) unavailability of blood and blood products and (iii) lack of protocol directed treatment. Apart from these, 4 of these grade IV liver injury patients had associated severe TBI, which may be a relative contraindication to operate in some centres as the outcomes are poor in patients with severe TBI with hypovolemic shock. Eight of the WSES grade IV injury patients were operated within an hour and the rest within the first 24 h. These delays in LMICs like ours are due to the overwhelming number of emergencies, shortage of human resources and lack of protocol adherence [32, 33].

### Limitations

Data regarding the patients requiring adjunctive procedure for management of liver injury were not recorded in this study. We do not have data regarding the cause of mortality in patients who were initially managed non-operatively. Morbidity of NOM was not recorded. The results of this study are generalisable to the urban university hospitals in India and perhaps the other similar university hospitals in LMICs.

## Conclusion

In this multicentre cohort of trauma patients from urban university hospitals in India, one third of those with a blunt trauma to the abdomen suffered a liver injury. Operative management was undertaken in less than one third of those with liver injury. Success rate of non-operative management of liver injury is comparable to other countries.

## Supplementary information

Additional file 1. WSES liver trauma classification [8]. (DOCX 14 kb)

## Data Availability

The data are available to whoever wants them by emailing the corresponding author or the last author (MGW). They can write their aim or objective, and then, the authors can decide if that study can be done without duplication of the work.

## Abbreviations

LMIC: Low- and middle-income country
WSES: World Society Emergency Surgery
OM: Non-operative management
OM: Operative management
TITCO: Towards Improved Trauma Care Outcomes
AAST: American Association of Surgery for Trauma
RTA: Road traffic accidents
TBI: Traumatic brain injury
SBP: Systolic blood pressure
ISS: Injury severity score
GCS: Glasgow Coma Scale
FAST: Focused Assessment with Sonography in Trauma
