# Supplementary material for: Management of liver trauma in urban university hospitals in India: an observational multicentre cohort study
Source: World J Emerg Surg. 2020 Oct 15;15:58. doi: 10.1186/s13017-020-00338-9 (PMC7560107; doi:10.1186/s13017-020-00338-9)
Supplement: Supplementary file 1 — WSES liver trauma classification [8]. (DOCX 14 kb) [file 13017_2020_338_MOESM1_ESM.docx]

**Additional File -1** WSES liver trauma classification[8]

|  | WSES grade | AAST | Hemodynamic |
| --- | --- | --- | --- |
| Minor | WSES grade I | I–II | Stable |
| Moderate | WSES grade II | III | Stable |
| Severe | WSES grade III  WSES grade IV | IV–V  I–VI | Stable  Unstable |
